# Occasional finding of neurological disorders during children hearing loss evaluation using the ABR

**DOI:** 10.1016/S1808-8694(15)30089-6

**Published:** 2015-10-19

**Authors:** Luiz Carlos Alves de Sousa, Luciano da Silveira Rodrigues, Marcelo Ribeiro de Toledo Piza, Denise Rezende Ferreira, Danielle Barbosa Ruiz

**Affiliations:** a PhD in Neurosurgery - Department of Surgery - Medical School of Ribeirão Preto, University of São Paulo. Professor of Otorhinolaryngology - Medical School of Ribeirão Preto - UNAERP. CEO - Pararella Otorhinolaryngology Association; b Resident physician - ENT Service - Pararella Otorhinolaryngology Association. Hospital da Sociedade Portuguesa de Beneficência, Ribeirão Preto, SP; c M.S. in Otorhinolaryngology, Medical School of Ribeirão Preto, University of São Paulo. MD of the Otorhinolaryngology outpatient ward of the Pararella Otorhinolaryngology Association, Ribeirão Preto, SP; d M.S Student - Department of ENT - UNICAMP, Physician, collaborator at the communications disorders outpatient ward - UNICAMP; e 3rd year Resident physician - Pararella Otorhinolaryngology Association, Ribeirão Preto, SP. Otorhinolaryngology Department - Pararella Otorhinolaryngology Association, Ribeirão Preto, SP. Electro Bonini clinical Center - Medical School - University of Ribeirão Preto - UNAERP

**Keywords:** abr, bera, neurological diseases, brainstem auditory evoked potentials

## Abstract

One of the most important applications of the Brainstem evoked response audiometry (ABR) is in the evaluation of hearing loss in children. Today the ABR is also indicated in the screening of cochleo-vestibular syndromes to detect retrocochlear lesions, to monitor patients in a coma (brain death), in monitoring the brainstem during skull base surgery, etc. Among the many BERA qualities, is its capacity to evaluate the neurophysiologic integrity of the auditory brainstem pathway. In doing so, sometimes while evaluating hearing function in children we are faced with ABR waves that suggest the presence of retrocochlear lesions (trace asymmetry, increased interpeak intervals), many times confirmed through image studies. These cases are seen as occasional findings of neurologic disorders during children hearing loss evaluation. In this study we report 2 cases of neurologic disorders diagnosed with the use of the ABR to evaluate hearing loss in children.

## INTRODUCTION

### A brief history

The auditory system neurophysiology and its means of assessment were restrict to audiology research labs until thirty years ago. With the progress in electronic equipment it became possible to develop systems for stimulation, capture, amplification, computation e recording the neurophysiologic activities of our hearing system that made it possible its clinical application. As a consequence, such equipment started to be produced in large scale, making auditory neurophysiology and its methods of assessment shift from highly sophisticated laboratories to the clinical settings. Thus, a new and fascinating chapter started in audiology: electrophysiology of hearing (audiometry of electrical response).

Electrical response audiometry is the generic denomination of the methods that allow us to study the bioelectrical phenomenon that occurs in the auditory system as a response to sound stimulus (evoked auditory potentials), from the inner ear all the way to the cerebral cortex. All these electrophysiological tests aim at detecting a bioelectrical activity related to an event (presentation of a sound signal and the mental activity triggered by changes in the sound signal characteristics). This bioelectrical activity is produced when the sound stimulus is transformed into electricity within the inner ear and in all the synapses that occur successively in the auditory pathway.

The development of computers able to individualize and mediate different responses made feasible the exclusive recording of neural potentials emitted by the auditory pathways. It is likely that the ultimate step towards the clinical use of evoked response audiometry was the work of Jewett, who in 1970, based on experimental studies in cats, described the presence of 4 waves that were related to specific sites or origins located in the brainstem, which follow a first potential, the action potential (wave I)1. Since then, evoked response audiometry has been increasingly used in the clinical setting, broadening the potentials of its application. Currently, hearing electrophysiological tests are indispensable tools for the assessment, diagnosis and monitoring of numerous neurotologic diseases. Among evoked potentials, it is possible that the brainstem evoked response audiometry (BERA) be the one most used in the clinical practice. It allows us to acquire the hearing system electrophysiological activity in the brainstem, mapping auditory synapses of auditory pathways from the cochlear nerve, cochlear nuclei, superior olivary complex (pons) all the way to the inferior colliculus (mesencephalus).

A series of seven waves (the components of the BERA traces are called waves or potentials) may be recorded from the forehead-lobule or mastoid leads during the first 12ms after a moderate sound stimulus. These waves, sequentially labeled with Roman numerals seem to represent successive tracts and/or auditory path synapses. Of these seven waves, the first five of them are the ones that interest us, and among them, waves I, III and V are the ones that offer us the most important parameters for BERA interpretation. Waves I and II are generated in the auditory nerve, wave III in the neurons that leave the cochlear nuclei complex2-4, waves IV and V in the lateral superior lemniscus and the major negative potential that follows wave V in the inferior colliculus depolarization.^5^

At present, ABR applications are increasingly more used, in investigating childhood hearing loss, the screening of cochleo-vestibular syndromes, the search for retro-cochlear lesions, in monitoring coma status (brain death), in monitoring the brainstem in skull base surgeries etc.

One of ABR’s quality is its very capacity of assessing the neurophysiologic integrity of brainstem hearing paths. We are able to compare stimulus progression speed (latencies) in both ears. Thus, sometimes, during the study of children’s hearing thresholds, we see ABRs that suggest the presence of retro-cochlear lesions in the auditory pathways (asymmetry of traces, increase in inter-peak intervals), often times proved by an image exam. It is an occasional finding of some neurologic disease when in search of children’s hearing thresholds.

In this present investigation we report 2 cases of neurologic diseases as occasional findings during the investigation of childhood hearing loss through the ABR.

## CASE REPORT

### Case 1

**Figure 1:**
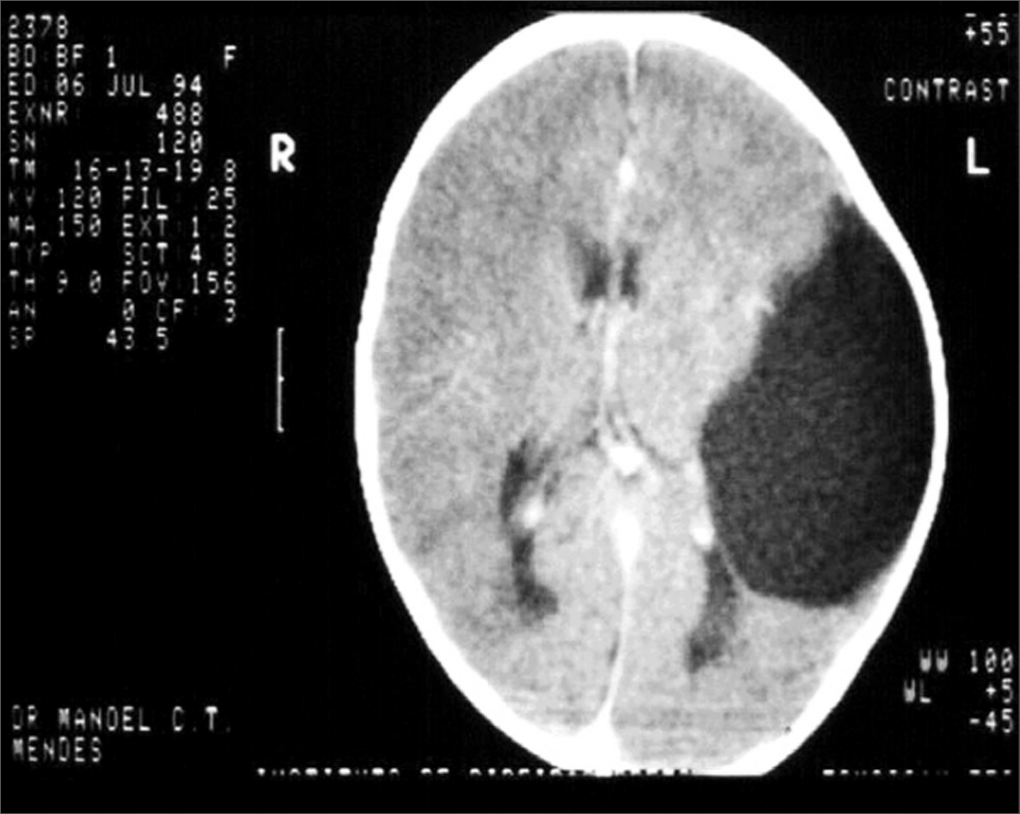
Notice the delay in waves III and V in the left ear.

**Figure 2:**
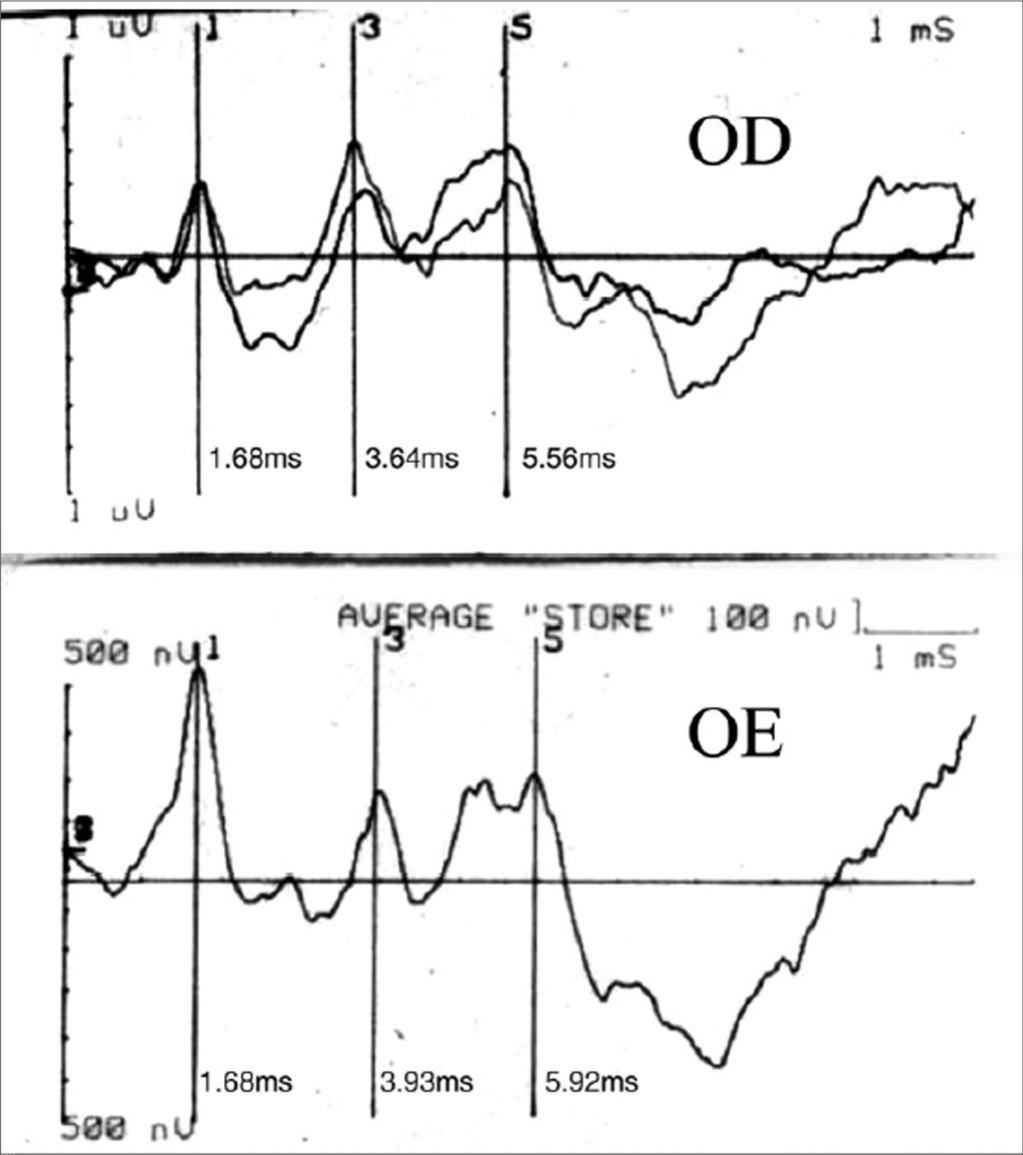
ABR’s suggestion of a retrocochlear lesion motivated us to order the image exam which showed an arachnoid cyst pushing the middle cerebral line and pushing the brainstem to the left.

B.N.C.C., 1 year and 11 months old, female, with suspicious auditory behavior. An electrocochleography showed objective bilateral thresholds of 30dBNA. Following with the investigation, the ABR revealed a wave V delay in the left ear, and also an increase in inter-peak intervals I-III, I-V, III-V, when compared to the right ear. The exam suggested conduction impairment at the left side brainstem. The suggestion of a retrocochlear lesion by the ABR motivated us to order an image study that revealed an arachnoid cyst pushing the cerebral mid-line and the brainstem to the left. It is an occasional finding of a neurologic disease when a child’s auditory threshold was investigated by using the ABR (Figures 1 and 2).

### Case 2

**Figure 3:**
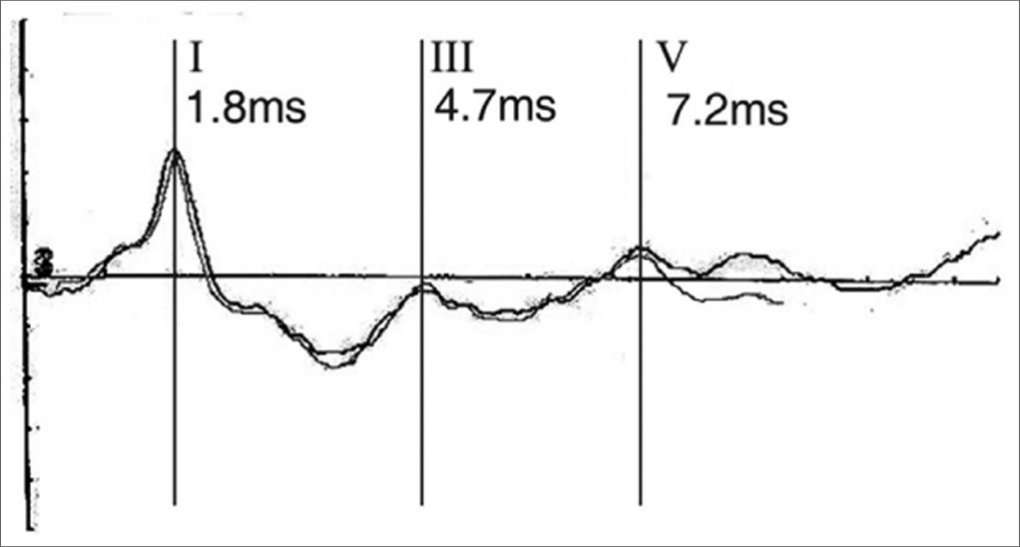
Notice the large increase in the LI-LV inter-peak interval and the low wave V amplitude.

**Figure 4:**
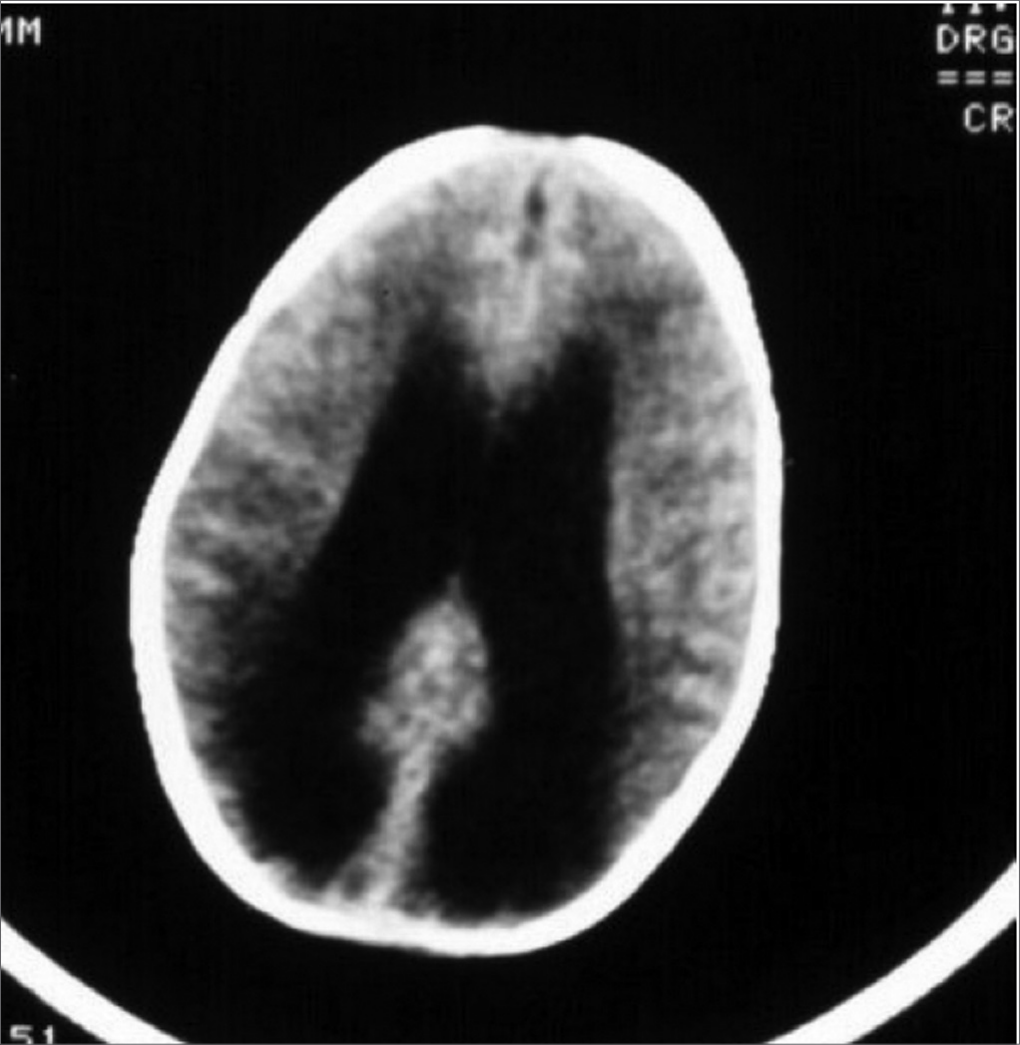
Important hydrocephaly seen by means of computerized tomography.

Y.S.F.A., 3 months, female. Was referred to hearing objective assessment. She had risk factors for hearing impairment. In her personal history, her mother reported seizures. The ABR revealed increase in inter-peak intervals I-V (5.4ms) and low amplitudes in waves III and V in both ears. Hearing thresholds were found to be moderately high in both ears. Her personal history coupled with the ABR’s finding led us to order an image study that revealed hydrocephaly (Figures 3 and 4).

## DISCUSSION

Recording brainstem auditory evoked potentials (ABR) allows us a glimpse of the physiology of this noble central nervous system segment (brainstem) that goes from the cochlear nucleus, going through the pons, all the way to the inferior colliculi (mesencephalus).

The ascending hearing pathways form numerous connections with central nervous system nuclei as part of a complex system of auditory reflexes. Intersections and internuclei communications make the auditory pathways occupy all the levels of the brainstem (middle and caudal portions of the pons and mesencephalus)^6^. This makes the ABR a non-invasive, reliable, objective and extremely useful test to monitor variations in the physiologic patterns of the brainstem auditory pathways.

Any disorders, degenerative, inflammatory, vascular, expansive or traumatic, that affect the brainstem would have to be extremely unique not to compromise the ABR’s wave synchronicity (neural element synchronicity)^7^, ^8^.

Understand the ABR’s physiologic principles broadens and enhances its clinical applications, for it is a test that detects and records brainstem and auditory nerve evoked potentials, the ABR indirectly provides us with information regarding the auditory pathway-related structures, for its proximity and through neural interaction mechanisms. Besides hearing investigation, numerous other applications have been described for the hearing pathways evoked potential test recently.

The investigation of retrocochlear pathologies, especially vestibular nerve schwannomas, illustrates such fact. For the anatomical proximity of structures within the inner ear and because these structures are encased in a bone tunnel, tumors and other inflammatory and expansive disorders of the vestibular and facial nerves may alter the electrical stimulus conduction through the auditory nerve, even if it is not a hearing alteration detected by some other method. Despite the fact that some authors question the ABR’s capacity to detect small retrocochlear tumors ^9^, other studies show that this test is very useful, detecting vestibular nerve schwannomas in 98% of the cases^10^.

In our experience with hearing electrophysiology, ABR has been able to differentiate cochlear alterations from those of the VIII nerve. We have used the ABR as a screening procedure to detect retrocochlear lesions such as vestibular schwannomas, other tumors in the posterior fossa (e.g. meningiomas), neuro-degenerative diseases (multiple sclerosis), vascular anomalies, etc.^11^, ^12^. For this end, the ABR is a test that bears exceptional sensitivity, especially for the early diagnosis of vestibular schwannomas (it is not as specific, though)^13^.

In a patient with hearing loss consequent to a cochlear disorder, stimuli of the same intensity presented to both, the diseased and the normal ear, will evoke waves V with latencies very close to one another. A cochlear lesion causes the phenomenon of electrophysiologic recruitment, in other words, we do not see, despite the hearing loss, any important delay in wave V latency in the affected ear, when compared to the contralateral ear. Actually, it is quite the contrary, sometimes we see wave V with lower latency in the affected ear, when compared to the normal ear.

**Figure 5:**
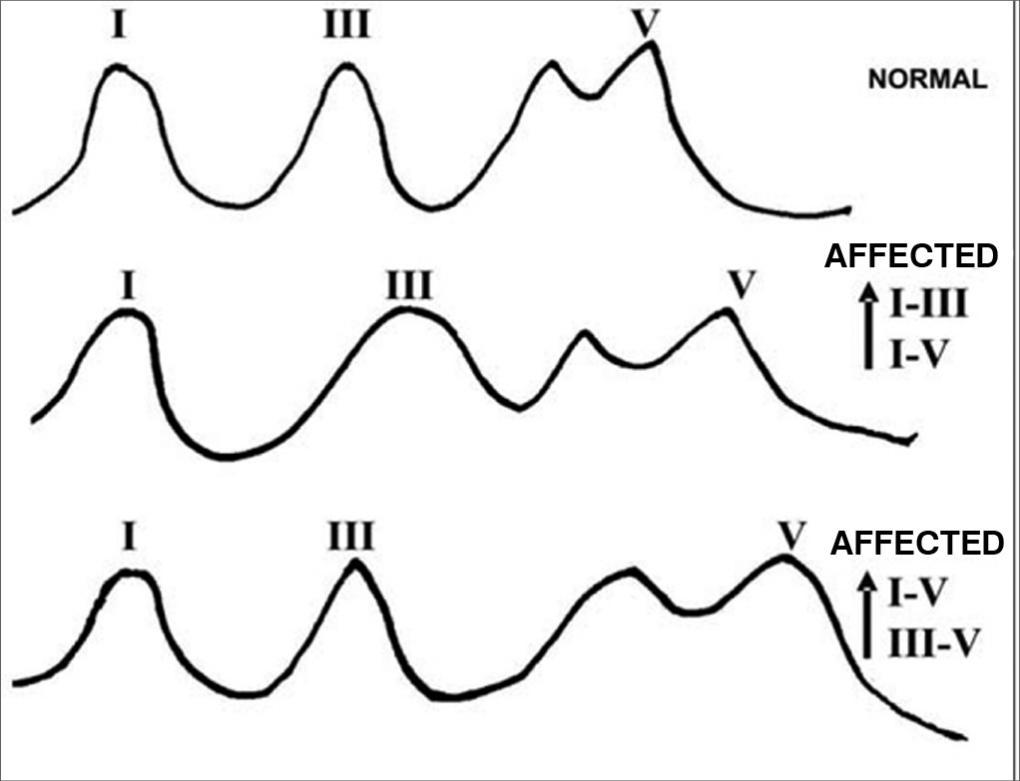
Examples of BERA traces inter-peak intervals in retrocochlear lesions.

In retrocochlear lesions we see major delays in wave V. Moderate hearing losses caused by such lesions may bring about delays of up to de 1.0ms, considered an eternity in short latency potentials electrophysiology (ECOG and ABR). Besides these delays in wave V, the ABR trace may present itself in other ways, as shown in Figure 5.

In our retrocochlear lesions screening protocol, the ABR traces in the affected ear, evoked by 90 dBHL stimuli, are considered as suggestive of retrocochlear lesions when the following characteristics are present:

• Wave V latency delay in the affected ear above 0,1ms for each dB of hearing loss (arithmetic average of psychoacoustic thresholds in the frequencies from 2 to 4 KHz), when compared to the wave V latency of the contralateral ear;
• Any wave V latency delays above 0,3ms (when compared to the contralateral ear) regardless of the hearing loss level in the affected ear;
• Presence of wave I only and absence of the others;
• Lack of all the waves in an ear with hearing loss below 70 dB (arithmetic average of psychoacoustic thresholds in the frequencies from 2 to 4 KHz).

The rigorous observation of ABR traces in the 2 aforementioned cases allowed us to suspect the presence of retrocochlear lesions in the auditory pathways, and through an image study we could prove the occasional finding of neurologic diseases when we were investigating children’s hearing thresholds.

## FINAL COMMENTS

ABR has proved to be an excellent exam for the investigation of hearing loss and hearing pathway disorders. However, proper knowledge about hearing electrophysiology and, consequently, the correct interpretation of the data obtained in the brainstem evoked potential survey will bring about accurate diagnosis, given by a broader view of the patient at hand. We presented two cases in which we encountered undiagnosed neurological disorders, through the investigation of hearing in children by means of the brainstem evoked response audiometry.
